# The sural-sparing pattern in clinical variants and electrophysiological subtypes of Guillain-Barré syndrome

**DOI:** 10.1055/s-0044-1785692

**Published:** 2024-04-19

**Authors:** Vinicius Furtado da Silva Castro, Roberto Teodoro Gurgel de Oliveira, João Daniel Lima dos Santos, Ramon de Souza Mendes, Agábio Diógenes Pessoa Neto, Emanuela Coriolano Fidelix, Mário Emílio Teixeira Dourado Júnior

**Affiliations:** 1 Universidade Federal do Rio Grande do Norte, Hospital Universitário Onofre Lopes, Departamento de Medicina Integrada, Natal RN, Brazil.; 2 Universidade Federal do Rio Grande do Norte, Departamento de Metemática, Natal RN, Brazil.

**Keywords:** Guillain-Barré Syndrome, Nerve Conduction Studies, Sural Nerve, Neurophysiology, Peripheral Nervous System Diseases, Síndrome de Guillain-Barré, Estudos de Condução Nervosa, Nervo Sural, Neurofisiologia, Doenças do Sistema Nervoso Periférico

## Abstract

**Background** Guillain-Barré syndrome (GBS) is the most common cause of acute flaccid paralysis worldwide and can be classified into electrophysiological subtypes and clinical variants.

**Objective** This study aimed to compare the frequency of the sural-sparing pattern (SSP) in subtypes and variants of GBS.

**Methods** This retrospective cohort study analyzed clinical and electrophysiological data of 171 patients with GBS hospitalized in public and private hospitals of Natal, Rio Grande do Norte, Brazil, between 1994 and 2018; all cases were followed up by the same neurologist in a reference neurology center. Patients were classified according to electrophysiological subtypes and clinical variants, and the SSP frequency was compared in both categories. The exact Fisher test and Bonferroni correction were used for statistical analysis.

**Results** The SSP was present in 53% (57 of 107) of the patients with acute inflammatory demyelinating polyradiculoneuropathy (AIDP), 8% (4 of 48) of the patients with axonal subtypes, and 31% (5 of 16) of the equivocal cases. The SSP frequency in the AIDP was significantly higher than in the axonal subtypes ( *p* < 0.0001); the value was kept high after serial electrophysiological examinations. Only the paraparetic subtype did not present SSP.

**Conclusion** The SSP may be present in AIDP and axonal subtypes, including acute motor axonal neuropathy, but it is significantly more present in AIDP. Moreover, the clinical variants reflect a specific pathological process and are correlated to its typical electrophysiological subtype, affecting the SSP frequency.

## Resumo

**Antecedentes** A síndrome de Guillain-Barré (GBS) é a causa mais comum de paralisia flácida aguda em todo o mundo e pode ser classificada em subtipos eletrofisiológicos e variantes clínicas.

**Objetivo** Este estudo teve como objetivo comparar a frequência do padrão de preservação do sural (SSP) em subtipos e variantes de GBS.

**Métodos** É um estudo de coorte retrospectivo que analisou dados clínicos e eletrofisiológicos de 171 pacientes com GBS internados em hospitais públicos e privados de Natal, Rio Grande do Norte, Brasil, entre 1994 e 2018. Todos os casos foram acompanhados pelo mesmo neurologista em centro de referência em neurologia. Os pacientes foram classificados de acordo com os subtipos eletrofisiológicos e variantes clínicas e a frequência do SSP foi comparada em ambas as categorias. O teste exato de Fisher e a correção de Bonferroni foram utilizados para análise estatística.

**Resultados** O SSP esteve presente em 53% (57 de 107) dos pacientes com polirradiculoneuropatia desmielinizante inflamatória aguda (PDIA), em 8% (4 de 48) dos pacientes com subtipos axonais e em 31% (5 de 16) dos casos não definidos. A frequência do SSP no AIDP foi significativamente maior do que nos subtipos axonais ( *p* < 0,0001); o valor manteve-se elevado após exames eletrofisiológicos seriados. Apenas o subtipo paraparético não apresentou SSP.

**Conclusão** O SSP pode estar presente na PDIA e nos subtipos axonais, incluindo a neuropatia axonal motora aguda, mas está significativamente mais presente na PDIA. Além disso, as variantes clínicas refletem um processo patológico específico e estão correlacionadas ao seu subtipo eletrofisiológico típico, afetando a frequência do SSP.

## INTRODUCTION

Guillain-Barré syndrome (GBS) is an autoimmune disorder that affects the peripheral nervous system. This disorder is the most common cause of acute flaccid paralysis worldwide, presenting a higher incidence among men and older adults. 1 The GBS diagnosis encompasses clinical presentation, electrodiagnostic features, and cerebrospinal fluid analysis. 2 3 4 5

The GBS can be classified according to demyelinating (i.e., acute inflammatory demyelinating polyradiculoneuropathy [AIDP]) and axonal subtypes (i.e., acute motor axonal neuropathy [AMAN] and acute motor and sensory axonal neuropathy [AMSAN]). 3 4 In addition, GBS can also be categorized into clinical variants, such as the classic sensorimotor, pure motor, paraparesis, pharyngeal-cervical-brachial, bilateral facial palsy with paresthesia, pure sensory, Miller Fisher syndrome (MFS), and Bickerstaff brainstem encephalitis. 5

The sural-sparing pattern (SSP), a useful tool for diagnosing GBS, is the sural sensory nerve action potential (SNAP) normal (or relatively spared) in the presence of abnormal median or ulnar SNAP. 6 This pattern was considered the most specific finding to differentiate AIDP from its mimics. 7 Additionally, Jin et al. 8 found SSP in the initial stages of the GBS. Previous studies associated SSP with AIDP 7 9 10 ; however, the pattern was also observed in MFS and axonal subtypes. 11 12 13 14 Moreover, SSP has several definitions in the literature that may hinder its analysis. 7 9 10 11

Therefore, this study aimed to compare the frequency of the SSP in electrophysiological subtypes and clinical variants of GBS.

## METHODS

This retrospective cohort study used clinical and electrophysiological data from medical records of 171 patients diagnosed with GBS hospitalized in public and private hospitals from Natal, Rio Grande do Norte, Brazil, between 1994 and 2018. The same neurologist (M. E. Dourado) followed the cases and conducted the neurophysiology evaluations. The study was conducted in the neurology center of the Integrated Clinical Medicine Department of the Onofre Lopes University Hospital of the Federal University of Rio Grande do Norte.

Patients were diagnosed with GBS according to Asbury and Cornblath 2 criteria. They were classified into AIDP or axonal subtypes (i.e., AMAN) according to electrophysiological criteria. 3 AMSAN is an acute axonal neuropathy involving motor and sensory fibers. The SNAP amplitude reduced by 50% in two nerves can predict the sensory involvement in axonal GBS and differentiate AMSAN from AMAN. 15 The cases that did not satisfy this criterion were classified as an equivocal subtype. Some patients were submitted to more than one electroneuromyography exam.

Median and ulnar sensory nerve responses were recorded antidromically with ring electrodes on the second and fifth fingers, respectively; surface stimulation was on the wrist, 14 cm proximal to the active recording electrode. Sural nerve potentials were recorded from the lateral malleolus with surface electrodes; the stimulation site was 14 cm proximal to the active recording electrode. Skin temperature of arms and legs was monitored and maintained above 32°C using a heater if needed. A sensory response was considered abnormal if values for the SNAP peak-to-peak (or baseline-to-peak) amplitude were below 6 uV in the sural nerve and 16 uV in the median nerve. 9

The analysis considered SSP as an absent median SNAP and present sural SNAP (criteria 1) or an abnormal median SNAP and normal sural SNAP (criteria 2). 9 We disregarded median SNAP changes attributable to carpal tunnel syndrome. The SSP frequency was compared between electrophysiological subtypes and clinical variants. When patients had two or more electrophysiological exams, the second data was considered for analysis, and these data were compared.

The clinical variants were classified into classic sensorimotor, pure motor, paraparetic, pharyngeal-cervical-brachial, bilateral facial palsy with paresthesias, pure sensory, MFS, and Bickerstaff brainstem encephalitis. 5 Patients with MFS who developed limb weakness were denominated MFS overlap with the GBS variant. The patients with acute ataxic neuropathy were classified as pure sensory or as an incomplete form of MFS according to the subtype. The demyelinating patients were classified as pure sensory, whereas those equivocal were classified as MFS. 16

### Statistical analysis

The proportion of patients with SSP was presented with its 95% confidence interval using the exact binomial method in each comparison. Then, proportions were tested for the H _0_ hypothesis of equality of proportions between groups using the exact Fisher test followed by Bonferroni correction for multiple comparisons. Moreover, the comparisons of electrophysiological subtypes and clinical variants were significant when *p* < 0.016 and *p* < 0.007, respectively. All analyses were performed in R ^®^ .

## RESULTS

Patients presented a mean age of 34.73 years; 60.8% (104) were men and 39.2% (67) women. The mean time from symptom onset to the first electrophysiological exam was 27.7 days; 15 patients did not have this information in the database.

The SSP was present in 38.6% (66 of 171) of the patients with GBS; 65.1% ( *n* = 43) did not have median SNAP and presented sural SNAP, and 34.9% ( *n* = 23) had abnormal median SNAP and normal sural SNAP. SSP frequency regarding the time to perform the early (up to seven days) or the late (over seven days) exam was seven (36.8%) and 59 (38.5%) days, respectively.

**Figure 1:**
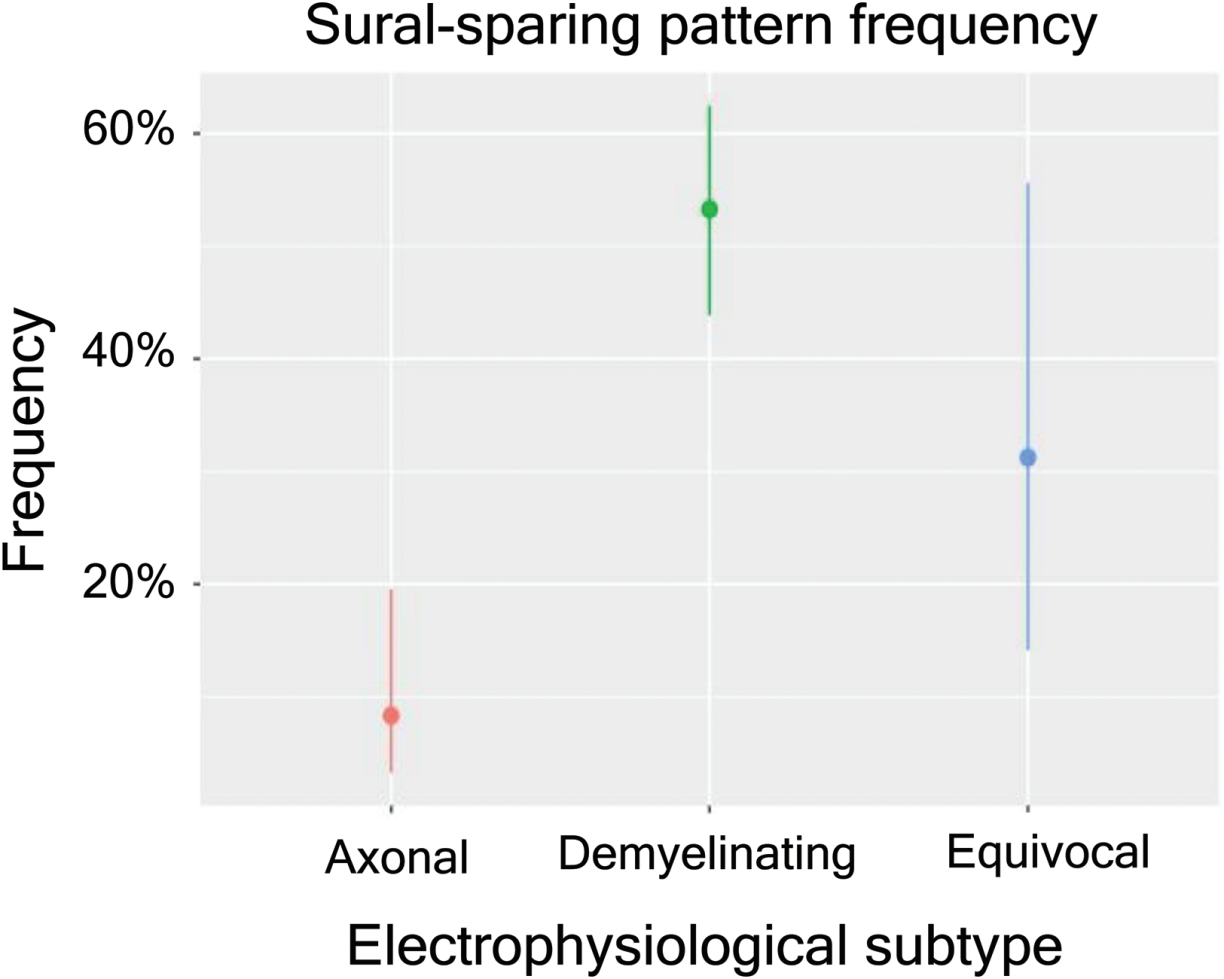
SSP frequency and its 95% confidence interval (CI) in the electrophysiological subtypes considering patients with at least one electromyography data.

Considering the electrophysiological subtypes ( Table 1 and Figure 1 ), 53.2% of the patients with AIDP and 8.3% with axonal subtypes presented SSP. Among the axonal subtypes, 4.9% of the patients with AMAN (2 of 41: case 1, sural SNAP of 11,4 uV and median SNAP of 13,9 uV; case 2, sural SNAP of 12 uV and median SNAP of 15,5 uV) and 28.5% with AMSAN (2 of 7: case 3, sural SNAP of 18,7 uV and median SNAP of 0 uV; case 4, sural SNAP of 5 uV and median SNAP of 0 uV presented SSP).

**Table 1:** SSP frequency and its 95% confidence interval (CI) in the electrophysiological subtypes considering patients with at least one electromyography data

Subtype	n	Sural sparing	Frequency	CI 95%	Demyelinating	Axonal
**Demyelinating**	107	57	0.53	(0.44, 0.62)	—	p < 0.0001
**Axonal**	48	4	0.08	(0.03, 0.2)	p < 0.0001	—
**Equivocal**	16	5	0.31	(0.14, 0.56)	0.1153	0.0364

**Figure 2:**
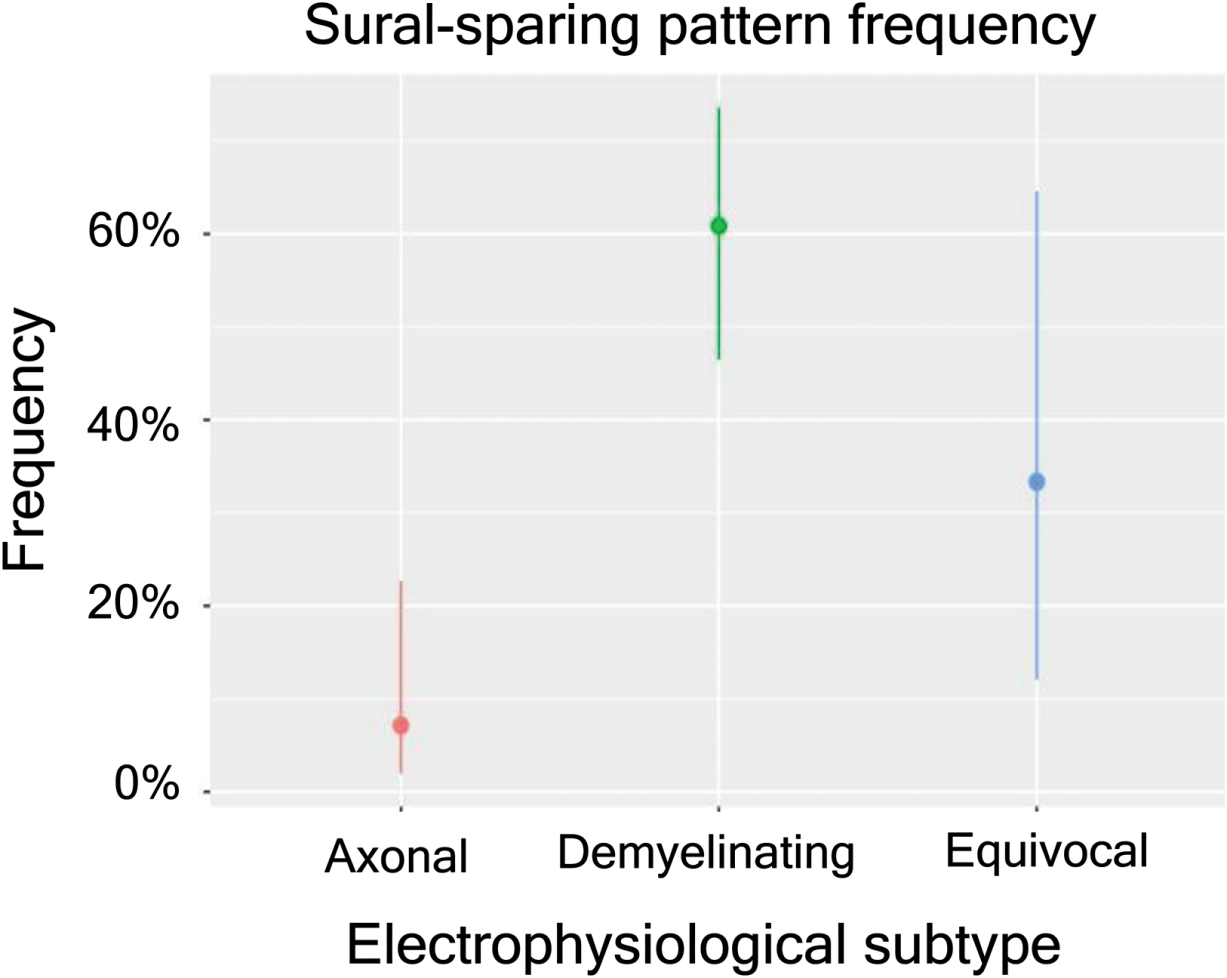
SSP frequency and its 95% confidence interval (CI) in the electrophysiological subtypes considering patients with two or more electromyography data.

Considering patients with two or more electromyography data, 39.7% (33 of 83) presented SSP ( Table 2 and Figure 2 ). The mean time from symptom onset to the second electrophysiological examination was 60.6 days; three patients did not present this information in the database.

**Table 2:** SSP frequency and its 95% confidence interval (CI) in the electrophysiological subtypes considering patients with two or more electromyography data

Subtype	n	Sural sparing	Frequency	CI95%	Demyelinating	Axonal
**Demyelinating**	46	28	0.61	(0.46, 0.74)	—	p < 0.0001
**Axonal**	28	2	0.07	(0.02, 0.23)	p < 0.0001	—
**Equivocal**	9	3	0.33	(0.12, 0.65)	0.1572	0.0812

**Figure 3:**
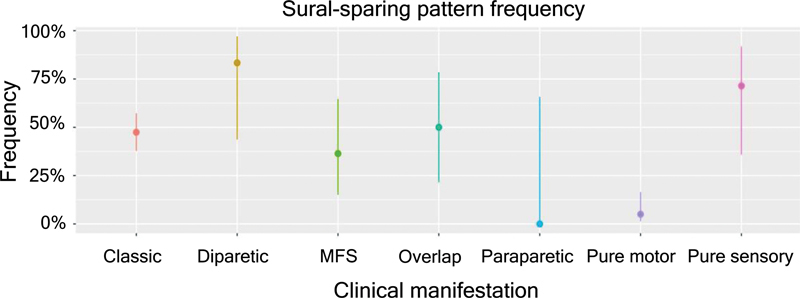
SSP frequency and its 95% confidence interval (CI) in the clinical variants.

Regarding the clinical variants ( Table 3 and Figure 3 ), the SSP was more frequent in bilateral facial palsy with paresthesia, classic sensorimotor, and MFS variants; the pattern was not observed in the paraparetic variant.

**Table 3:** SSP frequency and its 95% confidence interval (CI) in the clinical variants

Clinical variant	n	Sural sparing	Frequency	IC95%	Classic sensorimotor	Pure motor	Pure sensory	Miller-Fisher syndrome	MFS overlap with GBS	Paraparetic
Classic sensorimotor	97	46	0.47	(0.38, 0.57)	—	p < 0.0001	0.2651	0.5404	1	0.4974
Pure motor	40	2	0.05	(0.01, 0.17)	p < 0.0001	—	0.0003	0.0153	0.0046	1
Pure sensory	7	5	0.71	(0.36, 0.92)	0.2651	0.0003	—	0.3348	0.6084	0.1667
Miller-Fisher syndrome	11	4	0.36	(0.15, 0.65)	0.5404	0.0153	0.3348	—	0.6577	1
MFS overlap with GBS	8	4	0.5	(0.22, 0.78)	1	0.0046	0.6084	0.6577	—	0.4667
Paraparetic	2	0	0	(0, 0.66)	0.4974	1	0.1667	1	0.4667	—
Bilateral facial palsy with paraesthesias	6	5	0.83	(0.44, 0.97)	0.1123	p < 0.0001	1	0.1312	0.3007	0.1071

## DISCUSSION

The SSP was present in 38% of the patients with GBS, regardless of the electrophysiological subtype. The SSP was most frequently identified in the demyelinating subtype and the bilateral facial palsy with paresthesia variant; however, the pattern did not vary according to GBS duration.

The SSP is an electrophysiological pattern found in about one-third of the patients with GBS 7 9 10 11 ; it presents several definitions in the literature that may hinder its analysis. The SSP frequency in GBS has been attributed to its demyelinating pathology and is well documented only in AIDP. 9 10 Hiew and Rajabally 6 retrospectively applied different SSP definitions to patients with GBS, verifying important differences in sensitivity and specificity for AIDP. The authors concluded that historical definitions would be specific for the demyelinating subtype. On the other hand, Umapathi et al. 11 reported SSP in both axonal and demyelinating GBS. All previous criteria were based on motor conduction; however, Uncini et al. used SSP as a supportive for AIDP diagnosis. For axonal subtypes, this author included motor reversible conduction failure as suggestive of AMAN and sensitive reversible conduction failure plus SNAP reduction for AMSAN. According to the author, the probability for equivocal diagnosis was lower with these criteria compared with Hadden's and Rajabally's ones. 17

Nagappa et al. 18 reported SSP ranging from 10.5% to 84.5% depending on the criteria and in different GBS subtypes; the pattern was highly correlated with AIDP. The lowest frequency was noted when combining “absent median” or “absent ulnar” and “present” or “normal” sural, contradicting our results.

Even considering the historical definition proposed by Bromberg and Albers, 9 the present study observed SSP in demyelinating and axonal subtypes but with significantly higher frequency in patients with AIDP ( *p* < 0.0001); values were kept high after serial electrophysiological examinations ( *p* < 0.0001).

Abnormalities in studies about sensory nerve conduction are rather common and change over time. 18 SSP can be observed in the early stage of GBS and is not associated with a worse prognosis. 19 In the present study, SSP was present similarly in the early (up to seven days) (36.8%) and late (over seven days) (38.5%) exams; the pattern did not change over time.

Regarding axonal subtypes, the SSP was present in patients with AMAN and AMSAN, corroborating Umapathi et al. 11 results. In our study, two AMAN cases had minor sensory abnormalities, not achieving AMSAN criteria. By definition, AMAN patients should not have any sensory complaints or findings. However, Capasso et al. 14 demonstrated that sensory fibers are affected in AMSAN and AMAN subtypes using serial conduction studies.

In this sense, AMAN and AMSAN pathophysiology is related to the action of anti-ganglioside antibodies against the nodal axolemma and the paranodal region. These antibodies impair sodium channels, resulting in reversible conduction failure and axonal damage due to Wallerian-like degeneration in motor and sensory fibers. 20 21 Therefore, Yuki and Shahrizaila 22 suggest that AMAN and AMSAN are part of only one electrophysiological subtype and differ mainly by the extension of impairment, which is reinforced by the immunological profile they share. 23

Regarding clinical variants, this study suggested that they reflect the pathological process and are correlated to its electrophysiological subtype. Hence, the SSP frequency was expected to be higher in demyelinating and lower in the axonal variant. In the present study, SSP was significantly more frequent in the classic sensorimotor than in the pure motor variant ( *p* < 0.0001). All the patients with the pure sensory variant were classified as a demyelinating subtype, resulting in a higher frequency.

The SSP was present in 83% of patients with bilateral facial palsy with paresthesia, indicating its demyelinating nature. 24 Patients with the paraparetic variant did not have SSP, confirming its axonal pathophysiology. 25 The MFS associated with GBS presented similar SSP frequency compared with classic sensorimotor ( *p* = 1.00) and MFS ( *p* = 0.65) variants. This finding was expected because they share some pathophysiological components.

The SSP was present in 36% of the patients with MFS, a clinical variant that presents reduced action potentials of sensory nerves and abolished H reflexes. 26 The pathological process for the decreased SNAP in patients with MFS remains controversial. According to Sekiguchi et al., 13 this process may occur due to a dying-back Wallerian-like degeneration from neuronal injury at the dorsal root ganglion. However, Umapathi et al. 27 improved SNAP amplitude in patients with MFS. This finding was inconsistent with neuronal death and possibly reflected a reversible conduction failure and distal axonal degeneration from nodal and paranodal axolemma dysfunction, similar to the pathophysiology of AMAN and AMSAN. 12 Nevertheless, SSP frequency in MFS and demyelinating variants were similar.

Two main hypotheses may explain the SSP. The first is related to the higher susceptibility of median and ulnar nerves to entrapment, which could hamper the blood-nerve barrier and facilitate the attack by autoantibodies; in this case, the sural nerve may be spared. 11 28 The second is based on the preferential demyelination on distal nerve terminals and is related to the measurement sites on nerve conduction studies. Since median and ulnar nerves are examined distally, fewer effects of demyelination were expected on the sural nerve since it is examined in an intermediate portion to its terminal. 9 29

This study reinforces that the SSP may be present in demyelinating and axonal subtypes, including AMAN, but with significantly higher frequency in AIDP. Moreover, the clinical variants reflect a specific pathological process and are correlated to its typical electrophysiological subtype, affecting the SSP frequency. The definition criterion may also interfere with this frequency.

SSP should be investigated in patients with acute flaccid paralysis and, when present, be used to support the GBS diagnosis. To investigate SSP, besides the sural nerve, at least one other sensory nerve from the upper limb must be investigated, depending on the definition used.
